# Nasopharyngeal Dermoid Requiring a Unilateral Tonsillectomy at Day Three of Age

**DOI:** 10.7759/cureus.60349

**Published:** 2024-05-15

**Authors:** Richa S Nathan, Aaron Zlatopolsky, Lara K Reichert

**Affiliations:** 1 Otolaryngology - Head and Neck Surgery, Albany Medical Center/Albany Medical College, Albany, USA

**Keywords:** neonatal respiratory distress, hairy polyp, infant, nasopharynx, nasopharyngeal neoplasm, neonate, tonsillectomy, dermoid tumors

## Abstract

Nasopharyngeal dermoid tumors, or hairy polyps, are rare benign congenital malformations of bigerminal origin with both ectodermal and mesodermal elements. It is often seen in the neonatal period and can lead to respiratory distress and/or feeding disorders. Tonsillectomy is defined as a surgical procedure that completely removes the tonsil, including its capsule, by dissecting the peritonsillar space between the tonsil capsule and muscular wall. This case demonstrates a female who was born at Albany Medical Center with no gestational complications. She presented with respiratory distress and increased work of breathing. When examined, she was noted to have a mass that extruded from her oral cavity. The mass was identified as a rare nasopharyngeal dermoid tumor that was peduculated to the left palatine tonsil. Transoral surgery was performed successfully and resulted in the excision of the dermoid tumor and left palatine tonsil, relieving the patient of respiratory distress with no complications. This case documents the rare concurrence of a nasopharyngeal dermoid tumor attached to the left tonsil, indicating the youngest tonsillectomy to date at day three of age. This case subsequently highlights the importance of including dermoid tumors in the differential of neonates experiencing respiratory distress.

## Introduction

Dermoid tumors are rare benign congenital lesions of ectodermal and mesodermal elements that often affect the nasopharynx. They can lead to respiratory distress and/or feeding disorders in the neonatal period [1]. The clinical presentation depends on the site and the size of the lesion. This presentation is often described as a polypoid mass protruding through the mouth as “a second tongue” [2]. Dermoid tumors are histologically composed of stratified keratinized epithelium with cutaneous structures, such as hair and sebaceous glands [1]. As of 2012, there were only 170 nasopharyngeal dermoid tumors reported in the literature [3]. Tonsillectomy is defined as a surgical procedure that completely removes the tonsil, including its capsule, by dissecting the peritonsillar space between the tonsil capsule and muscular wall; this can be done with or without adenoidectomy [4]. Each year, over 500,000 cases are performed in children 15 years of age or younger [5]. The two most common indications for tonsillectomy are sleep-disordered breathing, causing respiratory distress, and recurrent tonsilitis. A retrospective study on 190 children younger than three years who underwent tonsillectomy showed an average age of two years and four months at the time of operation [6].

This report describes a complex and rare case of a benign nasopharyngeal dermoid or hairy polyp mass requiring surgical excision and subsequent tonsillectomy in a three-day-old patient. Based on the literature, this is the youngest patient to have ever undergone a tonsillectomy. This case emphasizes the importance of adding a nasopharyngeal dermoid tumor to a differential when a neonate is experiencing respiratory distress and work of breathing.

This article was previously presented as a meeting poster at the 2023 American Academy of Otolaryngology and Head and Neck Surgery Conference on October 2, 2023.

## Case presentation

**Figure 1:**
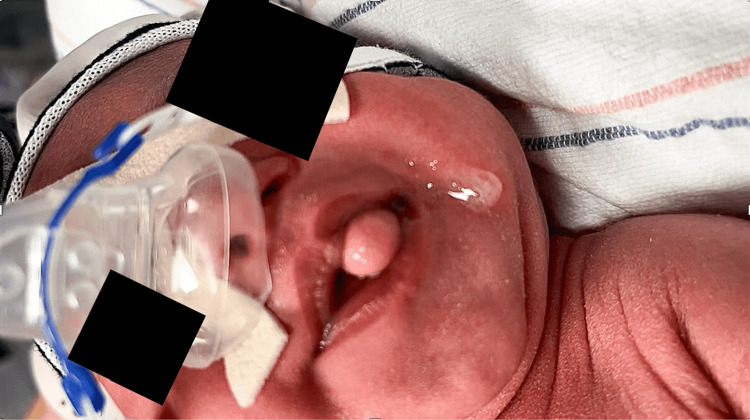
Nasopharyngeal dermoid tumor shown pre-operatively. The nasopharyngeal dermoid tumor is seen protruding through the mouth from the left oral cavity as a “tongue-like” mass.

A one-day-old female was transferred to Albany Medical Center due to respiratory distress. She was born at full term with no gestational complications. The patient was found to have increased work of breathing with oxygenation below 88%. Her respiratory status improved when she was placed on her side while on continuous positive airway pressure (CPAP). When examined, the patient was noted to have an oropharyngeal mass, which extruded from her oral cavity (Figure 1).

**Figure 2:**
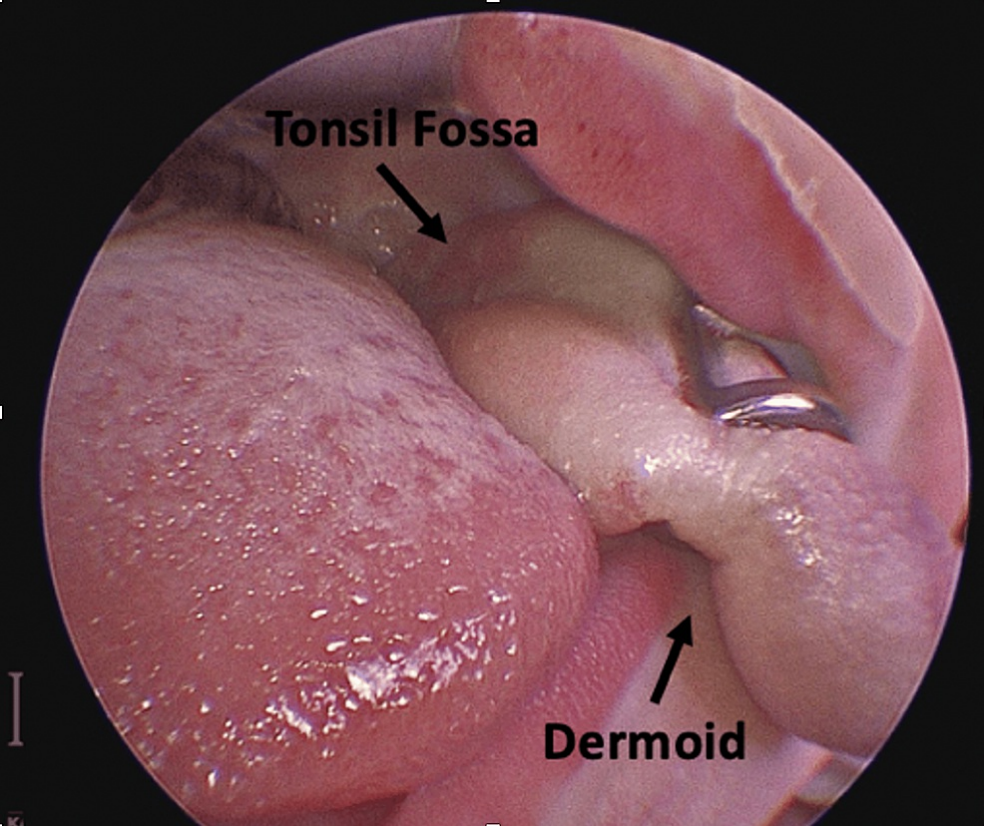
Preoperative image of the dermoid tumor. Shown is an intraoperative close-up image of the nasopharyngeal dermoid tumor from the left side of the patient's oral cavity. The orientation of the tonsil fossa with regard to the dermoid tumor is demonstrated with arrows.

The patient was subsequently taken to the operating room where an endoscopic evaluation was performed using a rigid Hopkins telescope. A mass with a tongue-like tissue appearance was observed to be emanating from the left pharyngeal wall, soft palate, and left palatine tonsil (Figure 2).

**Figure 3:**
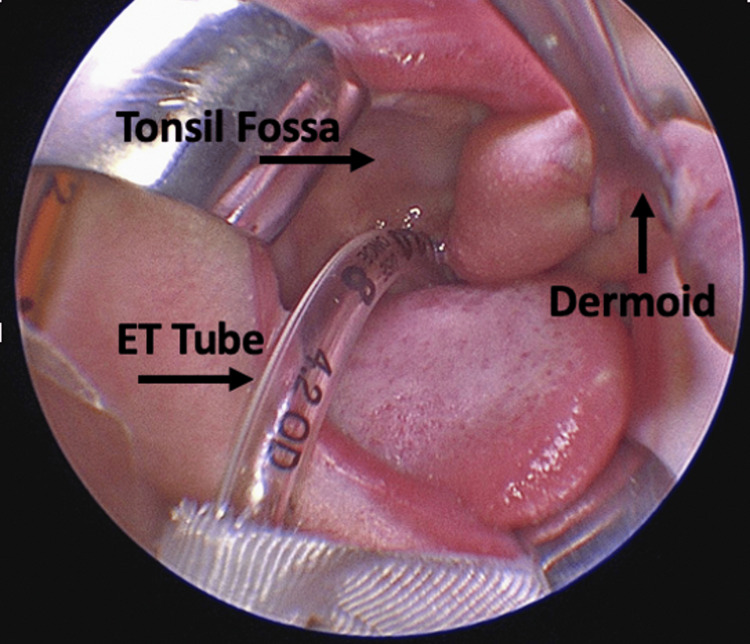
Intraoperative image of the dermoid tumor after endotracheal intubation. Shown is an image of the dermoid pedunculated to the left palatine fossa and endotracheal tube (ET tube) placement shown preoperatively. The orientation of the tonsil fossa with regard to the dermoid and endotracheal tube is demonstrated with arrows.

An endotracheal tube was placed to assist the patient with mechanical ventilation in preparation for the surgical resection of the nasopharyngeal dermoid tumor. The mass was noted to be adherent to the palatine tonsil, and the decision was made to excise the tonsil with the lesion (Figure 3). 

**Figure 4:**
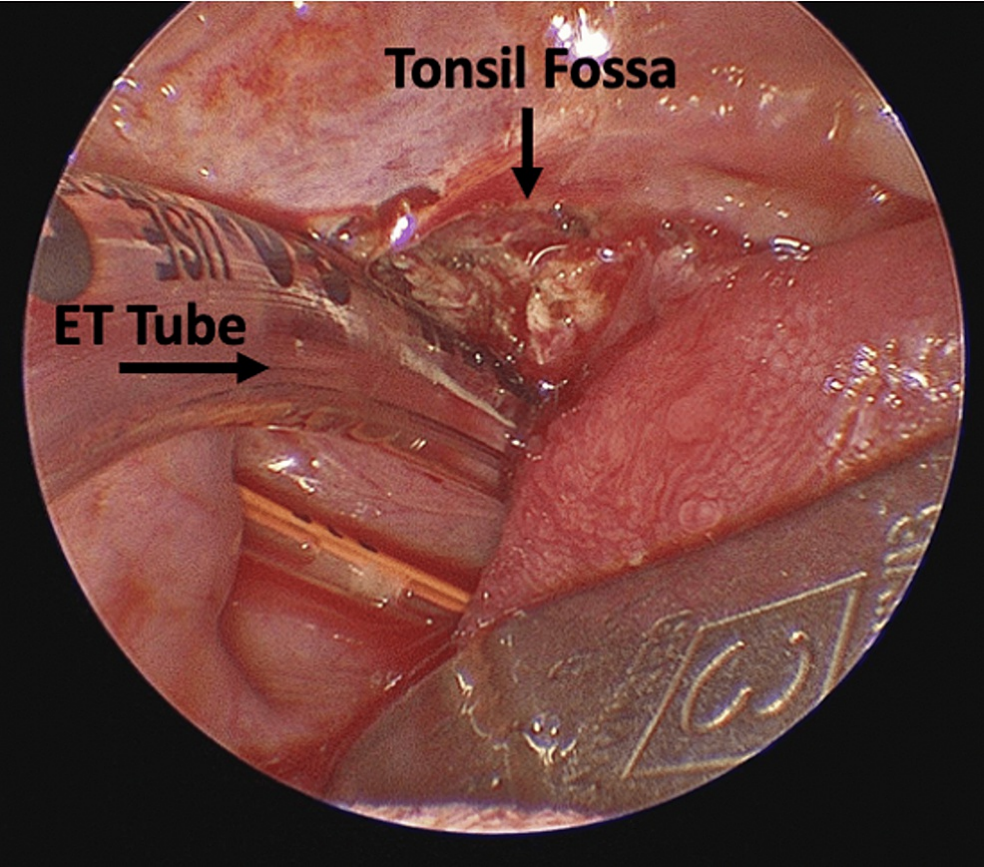
Tonsil fossa shown postoperatively. Shown is a postoperative image of the tonsil fossa after excision of the dermoid tumor and left palatine tonsil.

Bovie cauterization was utilized to excise the dermoid lesion from its attachments, including the left palatine tonsil. The patient tolerated the procedure well and did not require any further airway support; there was no blockage of the airway postoperatively (Figure 4). The mass was confirmed to be a benign nasopharyngeal dermoid tumor. 

**Figure 5:**
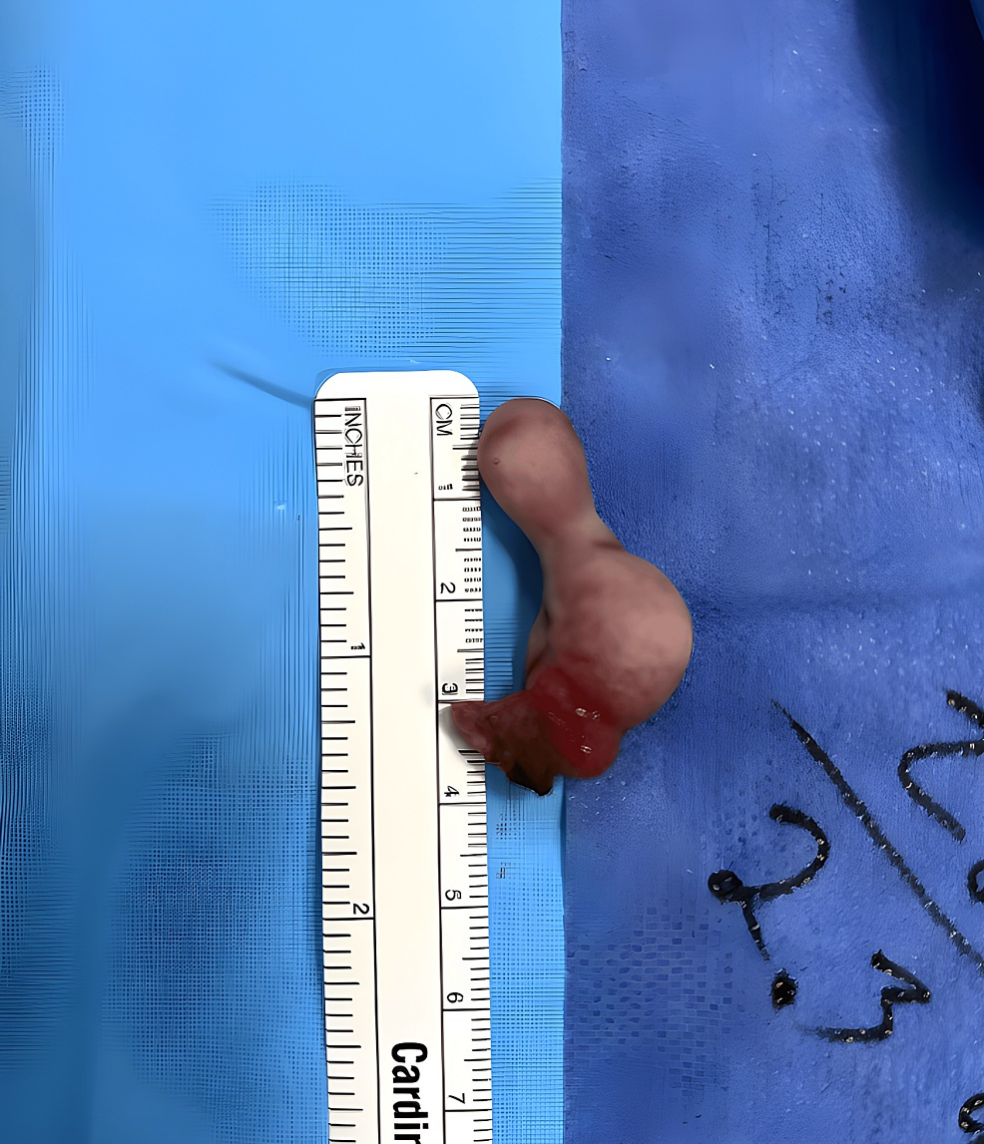
Dermoid tumor shown postoperatively. Shown is a gross image of the dermoid tumor post-excision, measured to be 4 cm in length.

After excision, the mass was measured to be 4 centimeters in length (Figure 5). 

## Discussion

The nasopharyngeal dermoid tumor was first cited in the literature in 1918 by Dr. A. Brown-Kelly in the *Journal of Laryngology, Rhinology, and Otology* at the Victoria Infirmary in Glasgow, Scotland [7]. Dr. Brown-Kelly's case discusses a six-week-old female who presented with an elongated growth, which first appeared at the age of one week; the pedunculated growth was attached to the left tonsil and tongue and caused severe respiratory distress. Upon its removal via a fine snare, the child's respiratory symptoms improved significantly and the mass was described to have a fleshy consistency with fine hairs; this patient presented similarly to our case. This case also reflected on the rarity and lack of reference to nasopharyngeal hairy polyps in the literature at that time, which still stands today [7].

In 1996, Bough et al. described a total of 35 reported cases of nasopharyngeal dermoid tumors in the *Journal of Pediatric Surgery* [8]. Their article emphasizes the importance of widespread knowledge of the possibility of a nasopharyngeal dermoid malformation in the medical field in order to facilitate early intervention and prevent significant morbidity in the neonate population [8]. As of 2012, only 170 cases of nasopharyngeal dermoid tumors had been reported in the literature, according to Yilmaz et al. [6]. The classic clinical presentation of these tumors, as presented in the literature and our case, is a visualization of a pedunculated lesion in the pharynx. This can lead to dramatic respiratory distress (Figure 1) and, in our case, resulted in a tonsillectomy. Dermoid tumors also occur six times more frequently in female patients and are more likely to attach on the left side [6].

It is important to investigate other congenital malformations if a lesion as such is present, although our case did not present with any associated issues. The differential diagnosis of a neonatal nasopharyngeal mass can include a teratoma, encephalocele, hemangioma, and thyroglossal or lingual duct cyst [5]. The management of a case as such focuses on securing the airway with endotracheal intubation followed by successful surgical excision (Figure 4). The prognosis for dermoid tumors is very good and usually results in complete recovery of the respiratory function [6].

## Conclusions

This report describes a complex and rare case of a benign nasopharyngeal dermoid tumor or hairy polyp in a three-day-old patient. The mass arose from the left palatine tonsil and other oropharyngeal structures, such as the lateral tongue. The dermoid was measured to be 4 cm in length and caused severe respiratory distress, reflected by an oxygen saturation below 88% and increased work of breathing. Bovie cauterization was utilized to successfully excise the dermoid lesion from its attachments, including the left palatine tonsil. This case emphasizes the importance of adding a nasopharyngeal dermoid tumor to a differential when a neonate is experiencing respiratory distress and work of breathing. Based on the literature, this is also the youngest patient to ever undergo tonsillectomy.
